# BTLA Expression on Th1, Th2 and Th17 Effector T-Cells of Patients with Systemic Lupus Erythematosus Is Associated with Active Disease

**DOI:** 10.3390/ijms20184505

**Published:** 2019-09-11

**Authors:** Christoph Oster, Benjamin Wilde, Christof Specker, Ming Sun, Andreas Kribben, Oliver Witzke, Sebastian Dolff

**Affiliations:** 1 Department of Nephrology, University Hospital Essen, University Duisburg-Essen, 45122 Essen, Germany; Christoph_Oster@web.de (C.O.); Benjamin.Wilde@uk-essen.de (B.W.); ming.sun@stud.uni-due.de (M.S.); Andreas.Kribben@uk-essen.de (A.K.); 2 Department of Rheumatology and Clinical Immunology, Kliniken Essen-Mitte, 45239 Essen, Germany; C.Specker@kem-med.com; 3 Department of Infectious Diseases, University Hospital Essen, University Duisburg-Essen, 45122 Essen, Germany; Oliver.Witzke@uk-essen.de

**Keywords:** SLE, BTLA, Costimulation, IFN-γ, IL-10, IL-17A

## Abstract

An imbalanced T-cell homeostasis plays an important role in the pathogenesis of systemic lupus erythematosus (SLE). Co-stimulatory and co-inhibitory molecules regulate T-cell differentiation, survival, and cytokine production. B- and T-lymphocyte attenuator (BTLA) is a co-inhibitory molecule which negatively regulates T-cell activation. The aim of this study was to investigate BTLA expression on regulatory and effector CD4^+^ T-cells in SLE patients with and without lupus nephritis (LN) during active and inactive disease. Therefore, peripheral blood of forty-one SLE patients and twenty-one healthy controls (HC) was phenotypically analyzed. Next, ex vivo stimulated T-cells were analyzed for the expression of BTLA on Th1-, Th2-, and Th17-effector cells by flow cytometry. Renal involvement was defined as biopsy-proven LN. Disease activity was assessed by SLE disease activity index (SLEDAI). Percentages of peripheral unstimulated BTLA^+^ CD4^+^ T-cells were significantly decreased in SLE patients with active disease. However, ex vivo stimulated Th1, Th2, and Th17 effector T-cells, expressed increased percentages of BTLA expression in active disease. In contrast, the BTLA expression on CD4^+^CD25^++^CD127^−^ regulatory T-cells was not significantly different. BTLA seems to be an important co-inhibitory molecule in the T-cell homeostasis of patients with systemic lupus erythematosus and crucial for disease activity.

## 1. Introduction

Systemic lupus erythematosus (SLE) is an autoimmune disease characterized by a disturbed immune balance of the regulatory and effector axis. This disbalance affects especially T- and B-cells and subsequently leads to the production of autoantibodies and tissue inflammation, in particular kidney injury which is a common severe organ manifestation [1]. The immune system developed various mechanisms to orchestrate the delicate balance between regulatory and effector cells to prevent deleterious activation. A crucial step for the B-cell T-cell interaction is the costimulation [2].

Therefore, B- and T-cells express costimulatory molecules on the surface. Receptor ligation provides a stimulatory (positive costimulation) or inhibitory (negative costimulation) signal. One of the best characterized inhibitory pathways in this cascade is the CD80/CD86-CTLA-4 pathway [3]. More recently, another inhibitory pathway (PD-1/PDL1) has been extensively studied and reported to be a promising target in various malignant diseases [4]. Remarkably, this interaction seems to be crucial also for the immune balance since blocking this pathway can result in autoimmune diseases [5].

Dysfunctional co-stimulation and co-inhibition promote the break of tolerance and are associated with autoimmunity. In previous studies, we reported that a defective co-inhibitory PD1/PDL-1 axis is associated with autoimmune diseases such as systemic lupus erythematosus and ANCA-associated vasculitis [6,7,8]. The B- and T-lymphocyte attenuator (BTLA) is also an inhibitory receptor of the Ig superfamily member that interacts with the herpes virus entry mediator (HVEM), a member of the TNFR family [9]. BTLA is a co-inhibitor expressed on a wide range of cells including B-cells, T-cells, follicular T-helper cells (Tfh), natural killer T-cells (NKT) and dendritic cells [10,11]. BTLA ligation with HVEM attenuates T-cell activation resulting in a reduction of T-cell proliferation and dampened immune response. In murine lupus model BTLA^−/−^ MRL-lpr/lpr mice developed a severe AIH-like disease and an exaggerated lymphocytic infiltration into inflamed kidneys [12].

However, BTLA as a co-inhibitor is scarcely studied in human autoimmune diseases and its role in disease pathogenesis of SLE remains unclear. The aim of this study was to investigate the expression of BTLA on T-cells and its role on the effector cell function of Th1, Th2, and Th17 cells in patients with systemic lupus erythematosus.

## 2. Results

### 2.1. The Percentages of CD4^+^ BTLA^+^ T-cells are Significantly Decreased in Active SLE

**Figure 1:**
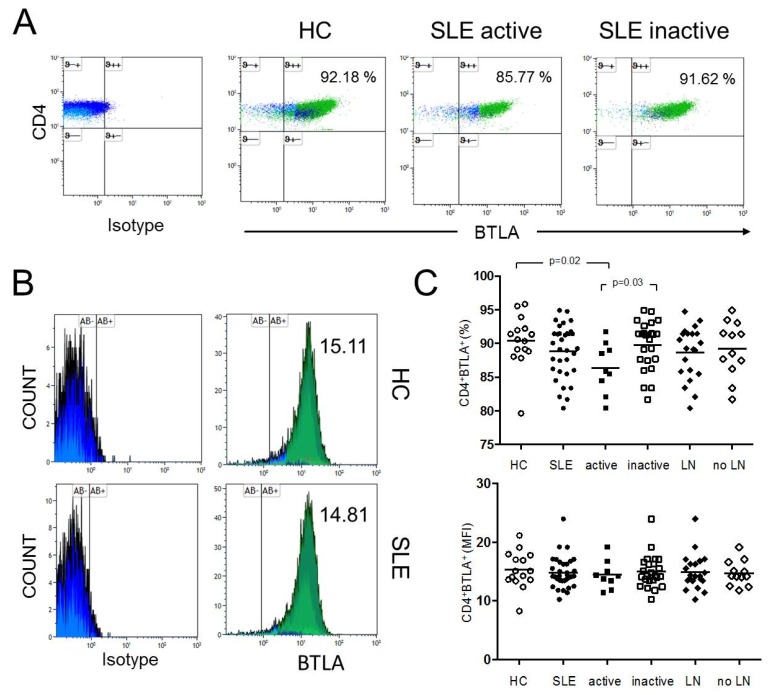
(**A**) This figure shows a representative dot plot of B- and T-lymphocyte attenuator (BTLA) expression on unstimulated CD4^+^ T-cells of a healthy control (HC) and of an active and inactive systemic lupus erythematosus (SLE) patient. (**B**) The histograms indicate the mean fluorescence intensity (MFI) of BTLA on unstimulated CD4^+^ T-cells of a HC and an SLE patient. (**C**) The summary of the results for HC (n = 15), all SLE patients (SLE, n = 33), active disease (active, n = 9), inactive disease (inactive, n = 24) and patients with lupus nephritis (LN, n = 21) and without LN (no LN, n = 12) is shown. Horizontal lines represent the mean values. *p*-values were calculated using the nonparametric Mann–Whitney U-test.

The expression of BTLA was studied in unstimulated CD4^+^ T-cells in SLE patients (n = 33) and healthy controls (n = 15) (Figure 1). The percentages of BTLA on CD4^+^ T-cells were significantly decreased in active SLE patients as compared to healthy controls (86.4% ± 3.8% vs. 90.4% ± 3.9%, *p* = 0.02) and as compared to inactive SLE patients, respectively (86.4% ± 3.8% vs. 89.8% ± 3.6%, *p* = 0.03). There was no significantly different expression of BTLA on CD4^+^ T-cells between healthy controls and inactive SLE patients (90.4% ± 3.9% vs. 89.8% ± 3.6%, *p* = 0.69). The mean fluorescence intensity (MFI) of BTLA on unstimulated CD4^+^ T-cells was 15.33 ± 3.13 in HC (n = 15) and 14.84 ± 2.68 in SLE patients (n = 33). There was no significant difference between these groups. The MFI of BTLA on unstimulated CD4^+^ T-cells of active SLE patients was not significantly different as compared to inactive SLE patients (14.46 ± 2.40 vs. 14.99 ± 2.81, *p* = 0.69).

### 2.2. Expression of BTLA is not Significantly Different on CD4^+^CD25^++^CD127^−^ Regulatory T-cells

**Figure 2:**
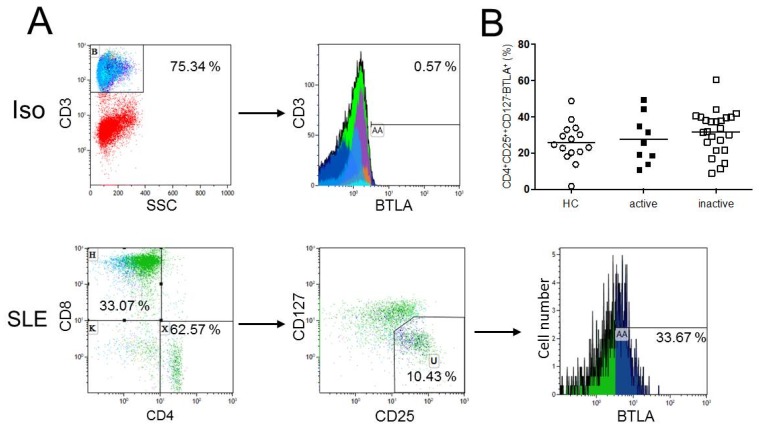
(**A**) This figure shows a representative flow cytometric data of BTLA expression on regulatory T-cells as defined as CD4^+^CD25^++^CD127^−^ T-cells of an SLE patient (SLE). The isotype control (Iso) is illustrated. (**B**) The histogram shows BTLA expression on CD4^+^CD25^++^CD127^−^ T-cells. There were no significant differences in the BTLA expression on CD4^+^CD25^++^CD127^−^ regulatory T-cells between healthy controls (n = 15), active (n = 9) and inactive (n = 24) SLE patients. Horizontal lines represent the mean values. *p*-values were calculated using the nonparametric Mann–Whitney U-test.

To determine the proportion of BTLA^+^ regulatory T-cells, the expression of BTLA on regulatory T-cells, defined as CD3^+^CD4^+^CD25^++^CD127^−^, was analyzed. There was no significant difference regarding BTLA expression on CD4^+^CD25^++^CD127^−^ regulatory T-cells of healthy controls (n = 15) as compared to SLE patients (n = 33) (25.8% ± 11.0% vs. 30.5% ± 12.2%, *p* = 0.20, Figure 2). There were also no significant differences between healthy controls and active SLE patients (25.8% ± 11.0% vs. 27.5% ± 13.4%, *p* = 0.91), healthy controls and inactive SLE patients (25.8% ± 11.0% vs. 31.7% ± 11.9%, *p* = 0.11). Active SLE patients as compared to inactive SLE patients were not different regarding the BTLA expression on CD4^+^CD25^++^CD127^−^ regulatory T-cells (27.5% ± 13.4% vs. 31.7% ± 11.9%, *p* = 0.41).

### 2.3. BTLA Expression on Th1, Th2 and Th17 Effector T-cells

**Figure 3:**
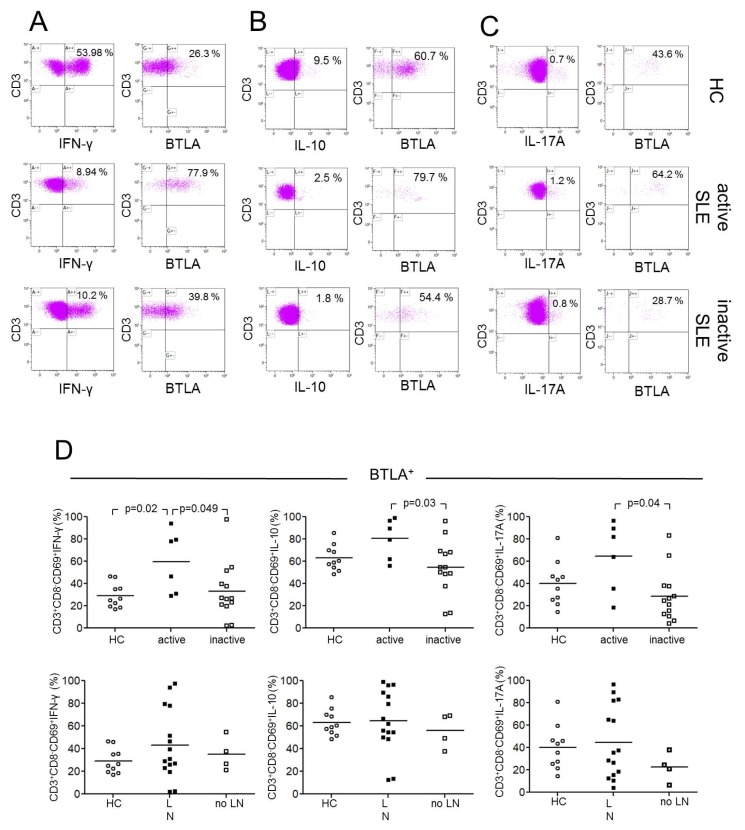
This figure shows representative dot plots of BTLA-expression on CD3^+^CD8^−^CD69^+^IFN-γ^+^(**A**), CD3^+^CD8^−^CD69^+^IL-10^+^ (**B**) and CD3^+^CD8^−^CD69^+^IL-17A^+^ (**C**) T-cells of a healthy control (HC), an active SLE patient (active SLE) and an inactive SLE patient (inactive SLE). (**D**) The expression of BTLA is shown on Th1-, Th2, and Th17-cells between healthy controls (HC), SLE patients with lupus nephritis (LN) and SLE patients without lupus nephritis (no LN). In the upper row open circles represent HC, filled squares represent active patients and open squares represent patients without inactive disease. In the lower row of this figure open circles represent HC, filled squares represent LN patients, and open squares represent patients without LN. Horizontal lines represent the mean values. *p*-values were calculated using the nonparametric Mann–Whitney U-test.

Next, we investigated the expression of BTLA on stimulated Th1, Th2, and Th17 effector T-cells characterized according to their lineage cytokines IFN-γ, IL-10, and IL-17 (Figure 3). The CD69 antigen is a type II integral membrane protein with a C-type lectin-binding domain and is widely accepted as an early activation marker. It is rapidly induced in activated lymphocytes and can be detected as early as 3–4 h following stimulation [13]. CD69^+^ T-cells were gated to detect activated T-cells, as described before [14]. We detected significant differences in the expression of BTLA in stimulated Th1-cells between healthy controls and active SLE patients (CD3^+^CD8^−^CD69^+^ IFN-γ^+^: %BTLA, 29.3% ± 11.0% vs. 59.7% ± 27.7%, *p* = 0.023) and in comparison of active versus inactive SLE patients (59.7% ± 27.7% vs. 33.0% ± 25.0%, *p* = 0.049, Figure 3D). There were no significant differences between healthy controls and inactive SLE patients (29.3% ± 11.0% vs. 33.0% ± 25.0%, *p* = 0.64).

Furthermore, there were significant different percentages of BTLA expression on stimulated Th2-cells between active and inactive SLE patients (CD3^+^CD8^−^CD69^+^IL-10^+^: %BTLA, 80.5% ± 18.0% vs. 54.4% ± 24.3%, *p* = 0.025, Figure 3D). There were no significant differences between healthy controls and active SLE patients (63.2% ± 11.5% vs. 80.5% ± 18.0%, *p* = 0.06) and between healthy controls and inactive SLE patients, respectively (63.2% ± 11.5% vs. 54.4% ± 24.3%, *p* = 0.25).

There were significant differences in expression of BTLA on stimulated Th17-cells between active and inactive SLE patients (CD3^+^CD8^−^CD69^+^IL-17A^+^: %BTLA, 64.4% ± 31.3% vs. 28.8% ± 23.1%, *p* = 0.039, Figure 3D). There were neither significantly different percentages in expression of BTLA between healthy controls and active SLE patients (40.2% ± 19.8% vs. 64.4% ± 31.3%, *p* = 0.12) nor between healthy controls and inactive SLE patients (40.2% ± 19.8% vs. 28.8% ± 23.1%, *p* = 0.13).

### 2.4. BTLA Expression on Th1, Th2 and Th17 Effector T-cells in Lupus Nephritis

The expression of BTLA on Th1, Th2, and Th17 effector T-cells was investigated in patients with and without lupus nephritis (Figure 3D). There was no significant difference in the expression of BTLA on stimulated Th1-cells in healthy controls as compared to SLE patients with lupus nephritis (29.3% ± 11.0% vs. 43.2% ± 31.0%, *p* = 0.23) nor compared to SLE patients without lupus nephritis (29.3% ± 11.0% vs. 35.0% ± 14.8%, *p* = 0.37). Additionally, the comparison of SLE patients with lupus nephritis with SLE patients without lupus nephritis (43.2% ± 31.0% vs. 35.0% ± 14.8%, *p* = 0.8) was not significant.

There was also no significant difference in the expression of BTLA on stimulated Th2-cells neither in comparison of healthy controls versus SLE patients with lupus nephritis (63.2% ± 11.5% vs. 64.4% ± 27.5%, *p* = 0.80) nor in the comparison of healthy controls versus SLE patients without lupus nephritis (63.2% ± 11.5% vs. 56.0% ± 15.2%, *p* = 0.45) nor the comparison of SLE patients with lupus nephritis versus SLE patients without lupus nephritis (64.4% ± 27.5% vs. 56.0% ± 15.2%, *p* = 0.52).

There was also no significant difference in the expression of BTLA on stimulated Th17-cells in healthy controls as compared to SLE patients with lupus nephritis (40.2% ± 19.8% vs. 44.7% ± 32.2%, *p* = 0.93) and in comparison of healthy controls versus SLE patients without lupus nephritis, respectively (40.2% ± 19.8% vs. 22.6% ± 12.9%, *p* = 0.11). The expression of BTLA on stimulated Th17-cells in SLE patients with lupus nephritis as compared to SLE patients without lupus nephritis was not significantly different (44.7% ± 32.2% vs. 22.6% ± 12.9%, *p* = 0.34).

### 2.5. BTLA Expression on Th1, Th2, and Th17 Effector T-cells is Independent of Treatment

**Figure 4:**
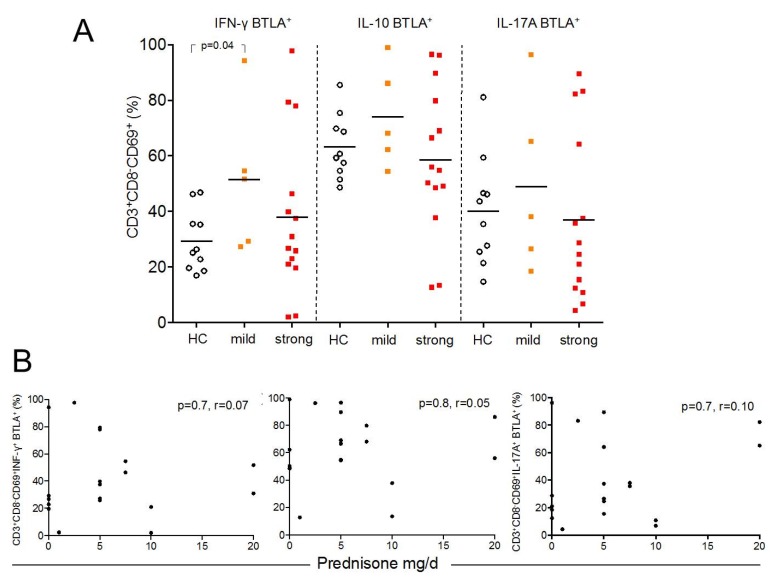
This figure shows the distribution of BTLA in Th1-, Th2-, and Th17-cells between healthy controls (HC, open circles), SLE patients with mild (orange filled squares) and strong (red filled squares) immunosuppression (**A**). The correlation between BTLA-expression and daily dose of prednisone in mg/d is illustrated in (**B**). Each measurement is represented as filled circle.

To assess the influence of immunosuppressive treatment on the percentages of BTLA expression on Th1, Th2, and Th17 effector T-cells we stratified the enrolled patients in one group with mild (n = 5) immunosuppression and another group with a strong immunosuppressive regimen (n = 14) (Figure 4A). There were no significant differences detected between these groups and healthy control for almost all effector subsets. Only SLE patients with mild immunosuppression had significantly more BTLA expression on Th1-cells as compared to healthy controls. Next, the percentages of BTLA expression on Th1, Th2, and Th17 effector T-cells were correlated with the daily dose of prednisone. There was no significant correlation detected (CD3^+^CD8^−^CD69^+^INF-γ^+^: %BTLA; *p* = 0.7, r = 0.07, CD3^+^CD8^−^CD69^+^IL-10: %BTLA; *p* = 0.8, r = 0.05, CD3^+^CD8^−^CD69^+^IL-17A: %BTLA; *p* = 0.7, r = 0.10) (Figure 4B).

## 3. Discussion

There is a growing body of evidence that co-stimulatory molecules play a crucial role in T-cell balance. These immune checkpoints seem to be important in the pathogenesis of malignancies and autoimmune diseases. The present study demonstrates that SLE patients with active disease have a decreased expression of the co-inhibitory molecule BTLA on CD4^+^ T-cells. Remarkably, active SLE patients express higher proportions of BTLA on IFN-γ, IL-10, and IL-17A producing effector cells. Regulatory T-cells have no different BTLA expression in SLE patients as compared to healthy controls.

A recent study by Sawaf et al. reported no significantly different expression of BTLA on CD4^+^ T-cells in SLE patients [15]. In line with these findings, we found no different mean fluorescence intensity of BTLA-expression on T-cells but significantly different proportions of BTLA-expression on T-cells. Interestingly, the authors demonstrated a defective upregulation upon T-cell stimulation with α-CD3/α-CD28 ex vivo. Moreover, they demonstrated an impaired BTLA functionality which is obviously due to poor recruitment of BTLA to the TCR cluster. The authors speculate that this mechanism is partially responsible for increased T-cell activation in SLE [15].

A murine autoimmune diabetes model has shown that mice transferred with BTLA^−/−^ OT-I cell showed aggressive insulitis and infiltration of islets suggesting that BTLA is required for regulation of CD8^+^ associated autoimmunity [16]. In a murine lupus model, CD4^+^ and CD8^+^ T-cells from BTLA^−/−^ MRL-lpr/lpr mice showed enhanced responses in α-CD3-induced proliferation as compared to BTLA^+/+^ MRL-lpr/lpr mice. CD4^+^ T-cells which secreted effector cytokines INF-γ, IL-10 and IL-17A expressed higher proportions BTLA^+^ in active SLE patients as compared to controls. BTLA-mediated, phosphatase-dependent signaling inhibits NF-κB activation thereby limiting the inflammatory T-cell response [17].

The present data suggest an upregulation of BTLA expression during active disease. Previous human SLE studies demonstrated a skewing of effector cells, in particular towards Th17 response [18,19]. In contrast to various costimulatory molecules (CD80, CD134 (OX40)) associated with an effector response towards IL-17 secretion, the proportion of BTLA expression was increased in all effector subsets studied during active disease [20]. This suggests that disease activity is related to BTLA upregulation.

**Figure 5:**
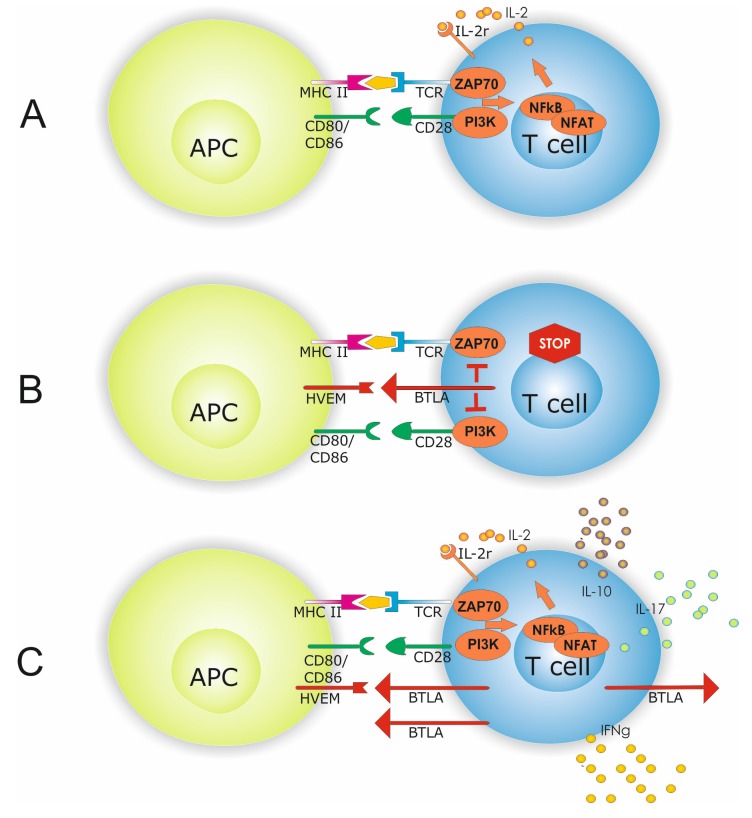
Antigen presenting cell (APC) dependent T-cell activation is shown in (**A**). APC T-cell interaction requires beside antigen presentation to the T-cell receptor (TCR) co-stimulatory molecules i.e., CD80/86 and CD28. Interaction of co-inhibitory molecules HVEM and BTLA inhibits intracellular signaling by limiting PI3K and ZAP70 activation which subsequently dampens T-cell hyperactivity (**B**). A hallmark of SLE is a disbalanced effector T-cell homeostasis with altered INF-γ, IL-10, and IL-17A expression. Increased expression of co-inhibitory molecules might indicate a counterbalance of this effector cell response (**C**).

Upregulation of BTLA during active disease might suggest a mechanism to counterbalance and co-inhibit this effector response during active disease (Figure 5). However, we did not test the inhibitory functionality of BTLA upregulation in SLE patients. Based on the data by Sawaf et al., it is conceivable that BTLA-mediated inhibition is less efficacious in SLE and upregulation of BTLA might thus be ineffective [15]. Interestingly, the degree of BTLA expression differed when comparing Th1, Th2, and Th17 cells. Th1 cells showed the lowest expression of BTLA in patients and HC, whereas Th2 cells showed the highest BTLA expression. This may explain the surprising finding that patients with active disease were not significantly different in terms of BTLA expression on Th2 and Th17 cells as compared to HC. If BTLA expression is already relatively high under physiological conditions, the potential for upregulation is limited. Thus, BTLA upregulation on Th2 and Th17 might be less pronounced and limited in magnitude in SLE patients. Therefore, significance is missed comparing BTLA^+^ Th2/Th17 cells of HC versus active SLE patients.

One might speculate that the counterbalance is less important in HC since BTLA expression is low. Moreover, the HVEM-expression status on antigen-presenting cells and the presence of soluble factors potentially binding BTLA on effector T-cells have not been investigated in the present study. Lacking this information, the interpretation is limited but this might be an additional explanation for different percentages of BTLA expression.

To assess the influence of renal involvement, we analyzed the results according to patients with and without biopsy-proven lupus nephritis. There was no significant difference. Additionally, we also could not find a relation with the level of immunosuppressive treatment.

In conclusion, co-inhibitory molecules came into focus of immunologic target therapies, in particular the CD80/CD86-CTLA-4 and the PD-1/PDL1 pathway. Novel molecules like BTLA^+^ are also pivotal for T-cell regulation and activation, especially in autoimmune diseases. The present study demonstrates that BTLA is expressed on various effector cells associated with disease activity in human SLE. Thus, further efforts should be made to elucidate the role of BTLA in SLE since checkpoint inhibition is an attractive therapeutic goal.

## 4. Materials and Methods

In this study, 41 SLE patients and 21 healthy controls were enrolled (Table 1). All patients fulfilled at least four of the American College of Rheumatology (ACR) criteria [18,19,20,21,22]. All patients provided informed consent and the study was institutional ethics committee of the University Duisburg-Essen (15-6323-BO, 21.07.2015). Thirty-nine of the 41 SLE patients and 19 of the 21 healthy controls were women. The mean age was 39.7 ± 13.5 years for SLE patients and 40.7 ± 13.9 years for healthy controls. Disease activity was assessed by SLEDAI (SLE Disease Activity Index). Active disease was defined as a SLEDAI score >4. Median SLEDAI Score was 2 (0–26). Renal involvement was defined as biopsy-proven lupus nephritis and present in 28 SLE patients. According to the RPS/ISN classification 2003, patients could be classified to class II (n = 8), class III (n = 4), class IV (n = 10) or class V (n = 5). One patient could not be classified. Except for five patients, all SLE patients received immunosuppressive treatment. Immunosuppressive treatment was classified in mild immunosuppression (prednisone and/or hydroxychloroquine) and strong immunosuppression. Strong immunosuppression included a combination of prednisone and/or hydroxychloroquine and azathioprine (n = 3) or mycophenolate mofetil (n = 10) or belimumab (n = 1).

In this study, two different experiments including whole blood staining (WBS) and ex vivo stimulation of peripheral blood mononuclear cells (PBMCs) were performed. For the WBS 33 of the 41 SLE patients with a mean age of 41.4 ± 13.3 years and 15 of the 21 healthy controls with a mean age of 44.7 ± 13.5 years were enclosed. For the ex vivo stimulation, 19 of the 41 SLE patients with a mean age of 36.1 ± 12.6 years and ten of the 21 healthy controls with a mean age of 31.1 ± 8.6 years were enclosed.

### 4.1. Antibodies

For the WBS, following antibodies were used in flow cytometry: Allophycocyanin (APC)-conjugated isotype (BD Pharmigen, Franklin Lakes, NJ, USA), phycoerythrin (PE)-conjugated isotype (BD Pharmigen, Franklin Lakes, NJ, USA), horizon violet 450 (HORV450)-conjugated anti-CD3 (BD Horizon, Franklin Lakes, NJ, USA), peridinin-chlorophyll-protein-complex (PerCP)-conjugated anti-CD4 (BioLegend, San Diego, California, USA), allophycocyanin H7 (APC-H7)-conjugated anti-CD8 (BD Bioscience, Franklin Lakes, NJ, USA), PE-conjugated anti-HLA-DR (eBioscience, Waltham, Massachusetts, USA), PE-conjugated anti-CD272 ( = BTLA1, BioLegend, San Diego, California, USA), PE-conjugated anti-CD279 ( = PD1, BD Pharmingen, Franklin Lakes, NJ, USA), APC-conjugated anti-CD270 ( = HVEM, BioLegend, San Diego, California, USA), FITC (fluorescein isothiocyanate)-conjugated anti-CD127 (eBioscience, Waltham, Massachusetts, USA) and PC7 (phycoerythrin-cyanine 7)-conjugated anti-CD25 (Beckman Coulter, Brea, California, USA).

Following antibodies were used for ex vivo stimulation of PBMCs: PE-conjugated isotype (BD Pharmigen, Franklin Lakes, NJ, USA), HORV450-conjugated anti-CD3 (BD Horizon, Franklin Lakes, NJ, USA), APC-H7-conjugated anti-CD8 (BD Bioscience, Franklin Lakes, NJ, USA), PE-conjugated anti-CD272 ( = BTLA1, BioLegend, San Diego, California, USA), phycoerythrin-cyanin 7 (PE-Cy 7)-conjugated anti-CD69 (Beckman Coulter, Brea, California, USA), FITC (Fluorescein isothiocyanate)-conjugated anti-interferon-γ (BD FastImmune, Franklin Lakes, NJ, USA), APC-conjugated anti-interleukin-10 (BioLegend, San Diego, California, USA) and PerCP-conjugated anti-interleukin-17A (BioLegend, San Diego, California, USA).

### 4.2. Immunophenotyping

First, 100 µL heparinized blood was mixed with antibodies anti-CD3 (HORV450), anti-CD4 (PerCP), anti-CD8 (APC-H7), anti-CD127 (FITC), anti-CD25 (PC7) anti-PD1 (PE), anti-HLA-DR (PE), anti-BTLA (PE), and anti-HVEM (APC). After vortex, all tubes were incubated for 30 min in the dark at room temperature. Next, 3 mL of VersaLyse™ was added in each tube and the suspension was mixed gently with vortex. Then, the tubes were incubated for 10 more minutes in the dark. Thereafter, the tubes were centrifugated and the supernatant was aspirated. The cell pellet was washed with 3 mL of phosphate-buffered saline (PBS). This washing step was repeated and finally 300 µl PBS were added before cells were immediately analyzed by flow cytometry.

### 4.3. Cell Isolation and Ex Vivo Stimulation

First, PBMCs were isolated from whole blood in sterile conditions with Lymphoprep™. After that, cells were stained with Tuerk’s solution and counted with Neubauer´s counting chamber. Then cells were frozen, as described before [23]. Briefly, PBMCs were suspended in FCS, Gibco^®^ RPMI 1640 medium and DMSO. The suspension was frozen in cryotubes at −80 °C overnight and then transferred to a nitrogen tank.

For stimulation, cells were gently thawed again. Cells were transferred from the nitrogen tank to a 37 °C water bath. In sterile conditions, the suspension from the cryotubes was transferred in a falcon tube with 4 mL of T-cell-medium which consists of Gibco^®^ RPMI 1640 medium + Gibco^®^ GlutaMAX™, FCS, Gibco^®^ Pen Strep, sodium pyruvate, and non-essential amino acids. Cells were washed and counted in a Neubauer´s counting chamber after coloring the cells with Trypan blue. The total number and the number of dead cells were defined to check the quality. Then, cells were concentrated or diluted for a concentration of two million cells per ml.

For stimulation purposes, two million cells were suspended in 2 mL T-cell medium and supplemented with 4 µL of eBioscience™ cell stimulation cocktail. Unstimulated samples served as controls. Cells were incubated for 4 h at 37 °C, 5% CO_2_. After incubation, the suspension was washed and suspended in PBS.

### 4.4. Staining

Anti-CD3 (HORV450), anti-CD8 (APC-H7), and anti-BTLA (PE) antibodies were added. After mixing the suspension, it was incubated for 30 min in the dark. Then, the cells were centrifuged and the supernatant was aspirated. Cytofix buffer was supplemented and gently mixed. After 20 min, incubation Perm/Wash™ buffer was added and washed twice. After adding 50 µL Perm/Wash™ buffer and 1 µL of mouse serum, the mixture was incubated for 10 min. at 4 °C. Anti-CD69 (PE-Cy 7), anti-interferon-γ (FITC), anti-interleukin-10 (APC), and anti-interleukin-17A (PerCP) antibodies were added to all tubes. After vortex, the suspension was incubated for 30 min at 4 °C. After washing, cells were analyzed immediately with a fluorescence-activated cell sorter (FACS) NAVIOS^TM^ from Beckman Coulter. Kaluza Analysis Software (Version 1.5, Beckman Coulter, Krefeld, NRW, Germany) was used for analysis of flow cytometric data. In the stimulated conditions, CD4^+^ cells were identified indirectly by gating CD3^+^ and CD8^−^ cells due to downregulated expression of CD4 on T-cells as described before [24].

### 4.5. Statistical Analysis

All values are expressed as mean ± standard deviation (SD). The nonparametric Mann–Whitney U-test was used to compare data from SLE patients with that of healthy controls. Spearman’s rank correlation was applied to detect correlations between different study parameters. Differences were considered statistically significant at a *p*-value < 0.05. GraphPad Prism 6.0 (GraphPad Software, Inc., California, USA) was used for statistical analysis.

## Figures and Tables

**Table 1:** Patients’ characteristics and immunosuppressive/immunomodulating therapy.

Characteristics	SLE Patients	HC	*p*-value
Total Number	41	21	
Women/Men	39/2	19/2	ns
Age (years, mean ± SD)	39.7 ± 13.5	40.7 ± 13.9	ns
SLEDAI (median (range))	2 (0–26)		
Active Patients (SLEDAI > 4)	12		
Inactive Patients (SLEDAI ≤ 4)	29		
Lupus Nephritis ISN/RPS Classification, n	28		
Class II	8		
Class III	4		
Class IV	10		
Class V	5		
Not Classified	1		
Treatment, n		21	
None	5		
Glucocorticoids, n	30		
*median dose (range), dose (mg/day)*	5 (1–80)		
Immunosuppressive/Immunomodulating, n			
Hydroxychloroquine	17		
*median dose (range), users (mg/day)*	400 (200–400)		
Methotrexate	1		
*median dose (range), users (mg/week)*	15		
Azathioprine	6		
*median dose (range), users (mg/day)*	75 (50–150)		
MMF	14		
*median dose (range), users (mg/day)*	1750 (500–2000)		
Cyclosporine A	2		
*median dose (range), users (mg/day)*	287.5 (250–325)		
